# Self-esteem and body image satisfaction in women with PCOS in the Middle East: Cross-sectional social media study

**DOI:** 10.1371/journal.pone.0301707

**Published:** 2024-04-25

**Authors:** Zainab Alkheyr, Mariam Murad, Priya Das, Khaled Aljenaee, Charlotte Kamel, Sulaiman Ali Hajji, John Flood, Stephen L. Atkin, Khawla F. Ali

**Affiliations:** 1 Royal College of Surgeons in Ireland-Medical University of Bahrain, Adliya, Bahrain; 2 Al-Adan Hospital, Kuwait City, Kuwait; King Abdulaziz University Faculty of Medicine, SAUDI ARABIA

## Abstract

**Background:**

Polycystic ovary syndrome (PCOS) is the most common endocrine disorder in women of childbearing age, associated with increased incidence of emotional disorders, anxiety and depression.

**Objective:**

The aim was of this study was to investigate whether those women reporting PCOS differed to women without PCOS in measures of psychological well-being including body-image satisfaction and self-esteem across a Middle Eastern population.

**Materials and methods:**

An online survey link of 21 questions was shared and distributed across social media platforms (Instagram and WhatsApp). The main outcome measured was levels of self-esteem and body image satisfaction in association with symptoms experienced by the participants.

**Results:**

12,199 female subjects completed the survey of whom 3,329 respondents (27.3%) self-reported a diagnosis of PCOS. PCOS respondents felt less attractive compared to non-PCOS respondents (73.9% vs 80.5%, p<0.0001). More respondents with PCOS reported avoidance of their reflection in the mirror (61.7% vs 49.8%, p<0.001) and avoidance of social interactions (22.3% vs 32.3%, p<0.0001). More PCOS respondents wanted to lose weight (75.2% vs 68.5%, p<0.001) with increasing weight being associated with being less attractive (p<0.001). Fewer PCOS respondents felt satisfied/confident compared non-PCOS respondents (38.6% vs 50.7%, p<0.001).

**Conclusion:**

PCOS respondents reported significantly lower measures of self-esteem and body image satisfaction compared to non-PCOS respondents in this population.

## Introduction

PCOS is a complex chronic disease with an overall prevalence of 6–10%, though this varies with ethnicity and it has been reported that the Middle Eastern prevalence of PCOS may be higher than in a Caucasian population [1,2]. Menstrual irregularity, anovulatory infertility and hirsutism are among the classic features of polycystic ovary syndrome (PCOS) on which the diagnostic criteria are based [3], such as the Rotterdam consensus, where after the exclusion of other conditions, two of three criteria; (i) oligo- anovulation, (ii) clinical and/or biochemical signs of hyperandrogenism and (iii) polycystic ovaries are required for the diagnosis. However, there is also an increase in the prevalence of metabolic features including type 2 diabetes (T2D), hypertension and cardiovascular disease [4], the causative mechanisms underlying this are unclear, though insulin resistance and obesity-related inflammation are thought to have major roles [4,5].

Within the 2023 international PCOS guidelines, there is the inclusion of body image as a new recommendation for the assessment in PCOS management [6], and a systematic review highlighted the body image concerns that women with PCOS have [7]. In a qualitative lived experiences in PCOS study poor mental health, dermatological and menstrual issues were highlighted, together with the report that more than half felt less feminine, and these also varied with ethnicity [8]. The term "body image" refers to a person’s perception of their body regarding their appearance, size, health, normal functioning, and sexual desires [9]. It is a complex concept that refers to a person’s perceptions and attitudes, as well as their feelings, thoughts, and actions, regarding their own body and appearance [10]. Body image and self-esteem develop in the context of sociocultural factors and attitudes towards the body of women with PCOS may vary across cultures, that would be in accord with a transnational study on non-PCOS body image [3]. A study reported differences between African-American and Caucasian women in body size perceptions, suggesting underlying differences in cultural attitudes toward weight [11]. Younger females were found to have higher concerns regarding weight and tend to be less satisfied with their bodies, and they are also heavily impacted by culture, media, and beauty standards [11]. An Iranian study reported that women’s self-esteem is solely based on their body image, which has an impact on their social activities and interpersonal relationships [12]. Women are more likely to be dissatisfied with their appearance when they do not conform to the social and cultural ideal of the female body that emphasizes on being thin [13]. The desire for the highly valued social acceptance by young women leads to excessive focus on appearance and basing their self-image and value heavily on their perceived beauty [13]. A systematic review in the USA reported that females with PCOS frequently experience higher levels of dissatisfaction with body image [14]. Extreme dissatisfaction from one’s body can lead to dysmorphic body disorder [15] which is characterised by unreasonable focus on imaginary or slight body defects. Because of importance put on outer appearance, people with dysmorphic disorder see themselves as unattractive and have a negative opinion of themselves [16].

In a recent study using the Body Appreciation Scale-2 (BAS-2) across 65 nations, 40 languages, gender identities, and age groups showed that the unidimensional BAS-2 model has widespread applicability [3]; however, there were large differences across nations and languages in latent body appreciation, while differences across gender identities and age groups were negligible-to-small, suggesting that studies in body image need to be population specific and may not be translatable to other ethnicities.

Social media provides healthcare providers with a mean to overcome barriers in delivering healthcare to patients [17], a tool for research [18] with widespread applicability [19]. The World Health Organization (WHO) and the Centres for Disease Control and Prevention (CDC) are examples of organizations that use such platforms to spread reliable information by providing daily updates and have thousands of followers all over the world [20]. As a result, social media demonstrates an impactful method of data collection and information delivery.

Many clinical features are associated with the emotional well-being of women with PCOS in the Arab region and the psychological effects of PCOS symptoms on their body image and self-esteem have received little attention in the MENA region; therefore, the aim was of this study was to investigate whether those women reporting PCOS differed to women without PCOS in measures of psychological well-being including body-image satisfaction and self-esteem across a Middle Eastern population.

## Materials and methods

### Study population

This was a descriptive cross-sectional study conducted among women following a social media platform with data collection between Jauary 22^nd^ until January 28^th^, 2023. The respondents were recruited conveniently using a web link that was accessible online throughout the study period through which users of the social media and messaging services Instagram and WhatsApp were invited to answer a survey with a series of multiple-choice questions that were collected and evaluated. The survey was created using Zoho Creator, an online tool from Zoho Corporation and was available in both English and Arabic.

### Survey administration & data collection

Female respondents who met the eligibility requirements for inclusion, which was 18 years of age or older and being an active social media user, were asked to complete a survey that included questions about the most common symptoms of PCOS. In addition, they were asked for information about their age, body mass index (BMI), body shape, marital status, comorbidities and whether they had PCOS. A self-guided, close-ended, and structured questionnaire was designed that consisted of 21 questions (see Supplemental material for questions) about body-image satisfaction and body confidence along with questions regarding their past medical history and demographical information. The questions captured the demographic characteristics, details on their PCOS symptoms and experience, menstrual cycle and scales related to study objectives such as self-esteem and body image satisfaction. The survey questions were from two published and validated surveys: Eating Disorder Examination Questionnaire (EDE-Q 6.0) [21] and the Adolescents Body Image Satisfaction Scale (ABISS) [22]. The questionnaires were adapted to compliment the local culture, making the questionnaire applicable and appropriate for the MENA region. The questionnaire was designed to take no longer than 10 minutes.

### Consent and ethical approval

Written informed consent was obtained from all participants for inclusion in the study. Written consent was obtained via a radio button before the questionnaire could be accessed. Questionnaires were submitted and anonymized. Ethical Committee approval was granted by Royal College of Surgeons in Ireland- Medical University of Bahrain (ethical approval number 05262022_398). Survey responses were entered through Zoho and collected on an excel sheet on a password-protected computer.

### Statistics

Statistical analysis was performed using SPSS version 26.0. Descriptive statistics were used to compute the frequencies and percentages for categorical data while the continuous data was expressed as means and standard deviations. The chi-square test was used to compute the significant differences between the categorical variables. Differences in continuous data between groups were calculated using Mann-Whitney U tests. Bivariate analysis was done using Spearman’s correlation method. Binary logistic regression methods were used to compute the odds ratio (OR) and to identify which variables were significantly associated with the outcome. All the tests were two-tailed and a p value of <0.05 was considered statistically significant.

## Results

A total of 12,199 female subjects completed the survey, whose mean age (SD) was 28.9 ± 6.4 years-old. A total of 3,329 respondents (27.3%, 95% CI: -29.7% to 84.3%) self-reported a diagnosis of PCOS (Table 1). Mean (SD) BMI for the PCOS respondents was 28.3±7.1 kg/m^2^ versus 26.7±6.9 kg/m^2^ in non-PCOS respondents (S1 Fig). For BMI category, more women with PCOS reported having obesity (p<0.001), and more women without PCOS were seen in underweight and normal categories (p = 0.002 & p< 0.001 respectively), but no differences were seen in overweight category (Table 1). For body shape category, more women with PCOS reported an inverted (p = 0.02) and rounded (metabolic) body shape (p<0.001), and more women without PCOS reported triangular body shape (p<0.01), but no differences were seen in the numbers of inverted, triangle, rectangle or hourglass body shapes (Table 1). For marital status, more women with PCOS reported being single (p<0.001). (Table 1).

**Table 1:** Demographics (all population).

	PCOS	Non- PCOS	p value
**Age (Mean**±SD, Yrs**)**	28.9 ± 6.4 (95% CI: 28.6–29.12)	31.2 ± 8.2 (95% CI: 31.03–31.37)	**<0.001**
**BMI (Mean**±SD, kg/m^2^**)**	28.35 ± 7.1 (95% CI: 28.06–28.54)	26.7 ± 6.9 (95% CI: 26.56–26.8428.9)	**<0.001**
**BMI Category** Underweight, N (%) Normal, N (%) Overweight, N (%) Obesity, N (%)	123 (3.7%) 1035(31.1%) 996 (29.9%) 1175 (35.3%)	444 (5.0%) 3518 (39.7%) 2647 (29.8%) 2261 (25.5%)	**0.002** **<0.001** 0.947 **<0.001**
**Body Shape** Inverted, N (%) Triangle, N (%) Rounded, N (%) Rectangle, N (%) Hourglass, N (%)	215 (6.5%) 868 (26.1%) 500 (15.0%) 684 (20.5%) 1062 (31.9%)	479 (5.4%) 2683 (30.2%) 824 (9.3%) 1967 (22.2%) 2917 (32.9%)	**0.03** **<0.001** **<0.001** 0.05 0.308
**Marital Status** Single, N (%) Married, N (%) Widowed, N (%) Divorced, N (%)	1708 (51.3%) 1465 (44.0%) 14 (0.4%) 142 (4.3%)	3699 (41.7%) 4639 (52.3%) 53 (0.6%) 479 (5.4%)	**<0.001** **<0.001** 0.273 **0.01**

A greater number of PCOS respondents had conception difficulty (19.9% vs 8.7%, 95% CI: -77.8 to 117.4% vs -33.6% to 51.1%, p<0.001), irregular periods (58.4% vs 23.9%,95% CI: -62.4% to 179.2% vs -40.1% to 87.8%, p = 0.001), hirsutism (70.1% vs 32.1%, 95% CI: -42.1% to 182.3% vs -38.0% to 102.1%, p = 0.001), acne (51.6% vs 36.8%, 95% CI: -70.8% to 173.9% vs -35.6% to 109.2%, p<0.001), male pattern hair loss (59.5% vs 37.3%, 95% CI: -60.8% to 179.2% vs -35.3% to 109.8%, p<0.001) and acanthosis nigricans (55.3% vs 35.3%, 95% CI: -66.1% to 176.9% vs -36.4% to 107.1%, p<0.001) (Table 2). In addition, women with PCOS reported a greater number of endocrine/metabolic and gynecological issues than women without PCOS (Table 3), though women without PCOS reported more hematological issues that were related to anaemia (reported as free text).

**Table 2:** Symptoms and PCOS.

	PCOS	Non-PCOS	p-value
**Conception difficulty** Yes, N (%) No, N (%)	661 (19.9%) 2663 (80.1%)	774 (8.7%) 8096 (91.3%)	**<0.001**
**Periods** Regular, N (%) Irregular, N (%)	1385 (41.6%) 1944 (58.4%)	6753 (76.1%) 2117 (23.9%)	**<0.001**
**Hirsutism** Yes, N (%) No, N (%)	2334 (70.1%) 995 (29.9%)	2847 (32.1%) 6023 (67.9%)	**<0.001**
**Acne** Yes, N (%) No, N (%)	1718 (51.6%) 1611 (48.4%)	3265 (36.8%) 5605 (63.2%)	**<0.001**
**Hair loss** Yes, N (%) No, N (%)	1980 (59.5%) 1349 (40.5%)	3306 (37.3%) 5564 (62.7%)	**<0.001**
**Acanthosis nigricans** Yes, N (%) No, N (%)	1840 (55.3%) 1489 (44.7%)	3128 (35.3%) 5742 (64.7%)	**<0.001**

**Table 3:** General systems questionnaire results.

	PCOS	Non-PCOS	p Value
**Respiratory Conditions** Yes No	18 (0.5%) 3311 (99.5%)	44 (0.5%) 8826 (99.5%)	0.434
**Metabolic/endocrine Conditions** Yes No	1096 (32.9%) 2233 (67.1%)	2471 (27.9%) 6399 (72.1%)	**<0.001**
**Cardiac Conditions** Yes No	1 (0.0%) 3328 (100.0%)	8 (0.1%) 8862 (99.9%)	0.460
**Hematology Conditions** Yes No	17 (0.5%) 3312 (99.5%)	109 (1.2%) 8761 (98.8%)	**<0.001**
**Gynecological Conditions** Yes No	3329 (100.0%) 0 (0.0%)	66 (0.7%) 8804 (99.3%)	**<0.001**
**Dermatological/Rheumatoid/orthopedic** **Conditions** Yes No	47 (1.4%) 3282 (98.6%)	124 (1.4%) 8746 (98.6%)	0.936
**Psychiatry Conditions** Yes No	11 (0.3%) 3318 (99.7%)	42 (0.5%) 8828 (99.5%)	0.353
**Other Conditions** Yes No	7 (0.2%) 3322 (99.8%)	24 (0.3%) 8846 (99.7%)	0.688

General systems answers were collated from patient’s free text answers.

Bivariate analysis and binary logistic regression analysis between BMI and PCOS showed that there was a significant positive correlation between increasing levels of BMI category and developing PCOS (r = 0.105, p<0.001), with greater odds of overweight and obese BMI categories being associated with PCOS [OR 1.36 (95% CI 1.5–2.3) and OR 1.87 (95% CI 1.5–2.3), p<0.005] respectively.

Features of attractiveness and self-esteem according to reported underweight, normal weight, overweight and obese BMI categories are shown in Table 4. In the underweight category of relatively few respondents, only “weight influences how you think about yourself as a person” appeared significantly different, but this did not differ after Bonferroni correction for multiple comparisons. In the normal weight category, more women with PCOS avoided seeing themselves in the mirror, avoided social interactions and fewer felt satisfied or confident (p<0.001) and those remained significant after Bonferroni correction for multiple comparisons. In the overweight and obese weight categories, women with PCOS scored less in both attractiveness and self-esteem (p<0.001) for all categories compared to women without PCOS subjects. There was no difference between women with PCOS and women without PCOS on wanting to lose, maintain or gain weight, nor was there a difference between groups when looking at the association with infertility.

**Table 4:** Features of attractiveness and self-esteem according to reported underweight, normal weight, overweight and obesity categories.

Underweight Category (n = 567)			
	PCOS	Non-PCOS	P value
**Weight influences how you think about yourself as a person** Yes No	83 (67.5%) 40 (32.5%)	250 (56.3%) 194 (43.7%)	**0.03** *
**Avoid seeing the mirror** Yes No	62 (50.4%) 61 (49.6%)	215 (48.4%) 229 (51.6%)	0.760
**Feel attractive** Yes No	97 (78.9%) 26 (21.1%)	360 (81.1%) 84 (18.9%)	0.607
**Avoid social interactions** Yes No	21 (17.1%) 102 (82.9%)	85 (19.1%) 359 (80.9%)	0.695
**Weight change** Lose Maintain Gain	9 (7.3%) 33 (26.8%) 81 (65.9%)	24 (5.4%) 143 (32.2%) 277 (62.4%)	0.431
**Satisfied/Confident** Yes No	69 (56.1%) 54 (43.9%)	289 (65.1%) 155 (34.9%)	0.073
Normal Weight Category (n = 4553)			
	PCOS	Non-PCOS	P value
**Weight influences how you think about yourself as a person** Yes No	698 (67.4%) 337 (32.6%)	2319 (65.9%) 1199 (34.1%)	0.370
**Avoid seeing the mirror** Yes No	535 (51.7%) 500 (48.4%)	1501 (42.7%) 2017 (57.3%)	**<0.001**
**Feel attractive** Yes No	837 (80.9%) 198 (19.1%)	2954 (84.0%) 564 (16.0%)	**0.02** *
**Avoid social interactions** Yes No	185 (17.9%) 850 (82.1%)	452 (12.8%) 3066 (87.2%)	**<0.001**
**Weight change** Lose Maintain Gain	419 (40.5%) 457 (44.2%) 159 (15.4%)	1481 (42.1%) 1516 (43.1%) 521 (14.8%)	0.647
**Satisfied/Confident** Yes No	631 (61.0%) 404 (39.0%)	2381 (67.7%) 1137 (32.3%)	**<0.001**
Overweight Category (n = 3643)			
	PCOS	Non-PCOS	P value
**Weight influences how you think about yourself as a person** Yes No	834 (83.7%) 162 (16.3%)	2065 (78.0%) 582 (22.0%)	**<0.001**
**Avoid seeing the mirror** Yes No	504 (50.6%) 492 (49.4%)	1096 (41.4%) 1551 (58.6%)	**<0.001**
**Feel attractive** Yes No	777 (78.0%) 219 (22.0%)	2170 (82.0%) 477 (18.0%)	**0.007**
**Avoid social interactions** Yes No	274 (27.5%) 722 (72.5%)	569 (21.5%) 2078 (78.5%)	**<0.001**
**Weight change** Lose Maintain Gain	918 (92.2%) 76 (7.6%) 2 (0.2%)	2363 (89.3%) 263 (9.9%) 21 (0.8%)	**0.012** *
**Satisfied/Confident** Yes No	378 (38.0%) 618 (62.0%)	1248 (47.1%) 1399 (52.9%)	**<0.001**
Obesity Category (n = 3436)			
	PCOS	Non-PCOS	P value
**Weight influences how you think about yourself as a person** Yes No	1059 (90.1%) 116 (9.9%)	1902 (84.1%) 359 (15.9%)	**<0.001**
**Avoid seeing the mirror** Yes No	560 (47.7%) 615 (52.3%)	872 (38.6%) 1389 (61.4%)	**<0.001**
**Feel attractive** Yes No	750 (63.8%) 425 (36.2%)	1658 (73.3%) 603 (26.7%)	**<0.001**
**Avoid social interactions** Yes No	594 (50.6%) 581 (49.4%)	871 (38.5%) 1390 (61.5%)	**<0.001**
**Weight change** Lose Maintain Gain	1156 (98.4%) 13 (1.1%) 6 (0.5%)	2212 (97.8%) 35 (1.5%) 14 (0.6%)	0.533
**Satisfied/Confident** Yes No	208 (17.7%) 967 (82.3%)	581 (25.7%) 1680 (74.3%)	**<0.001**

* not significant after Bonferroni correction for multiple comparisons.

**Fig 1:**
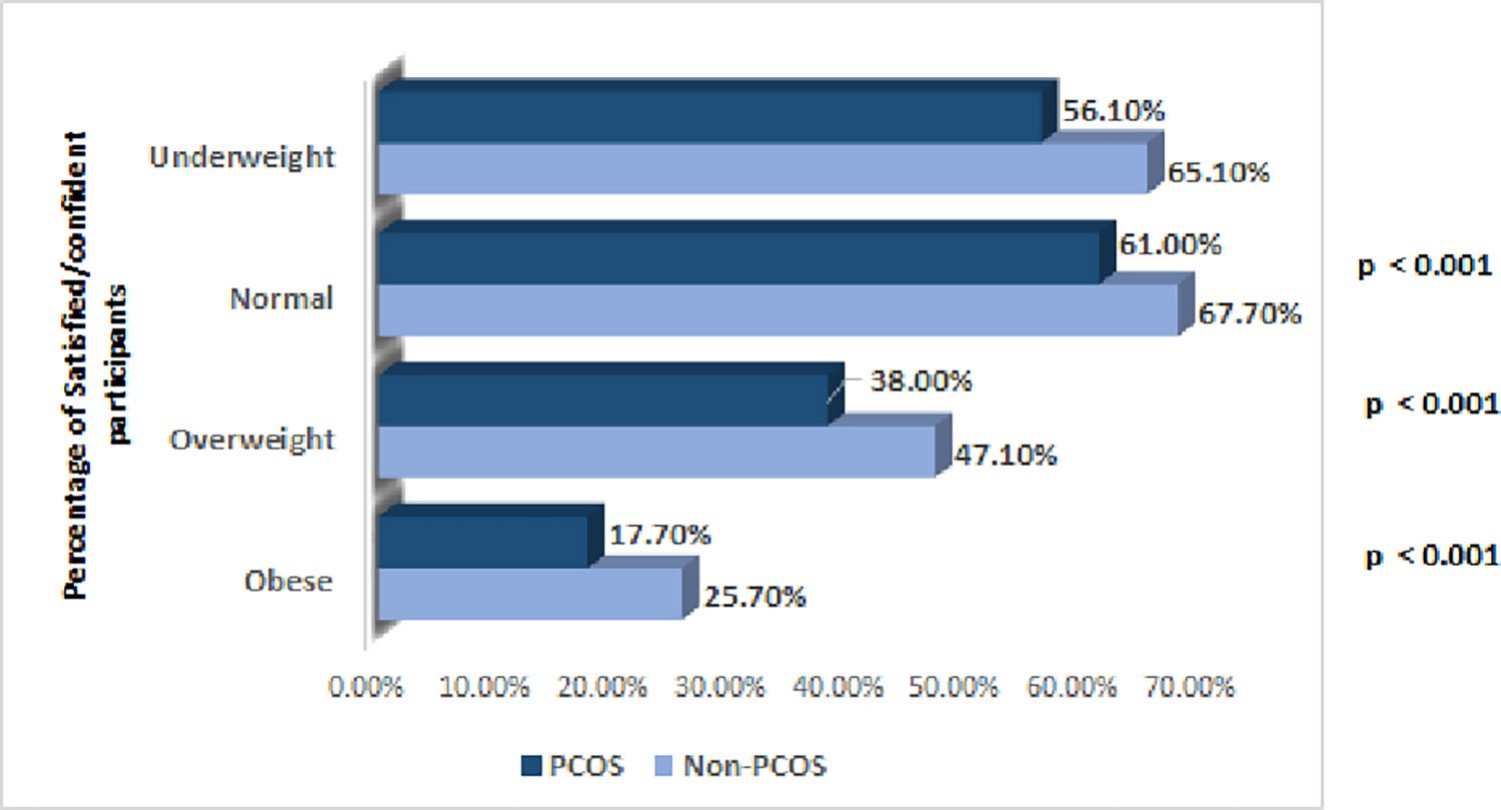
Percentage of satisfied/confident participants, with PCOS subjects vs non-PCOS subjects.

Overall, a significant difference was noted in the number of respondents who felt satisfied/confident in their physical appearance among PCOS respondents to that of non-PCOS respondents (38.6% vs 50.7%, 95% CI: -80.6% to 157.7% vs -24.3% to 125.7%, p<0.001). Fig 1 shows the percentage of satisfied/confident participants, with women with PCOS scoring less than women without PCOS subjects in all weight categories (difference between women with PCOS and women without PCOS; underweight (-9.0% ± 9.8%, 95% CI [-0.19, 0.0083], p = 0.07); normal weight -(6.7% ± 3.3%, 95% CI [-0.1, -0.034], p<0.001); overweight (-9.2% ± 3.6%, 95% CI [-0.13, -0.056], p< 0.001); obese (-8.0% ± 2.8%, 95% CI [-0.11, -0.052], p<0.001). Among the rounded body types, 500 respondents reported PCOS of whom 409 (81.8%) were more dissatisfied or less confident in comparison to their 601 women without PCOS respondents (72.9%) (8.9% ± 4.5%, 95% CI [0.043, 0.13], p<0.001). Similar trends were also seen for women with PCOS with a greater number reporting acne (12.0% ± 2.8%, 95% CI [0.09, 0.15], p< 0.01), clinical hirsutism (8.7% ± 2.7%, 95% CI [0.059, 0.11], p = 0.002) and hair loss (10.0% ± 2.7%, 95% CI [0.076, 0.13], p < 0.01) being dissatisfied as comparison to women without PCOS.

Binary logistics regression analysis was conducted to assess if PCOS, high BMI and body shape had an impact on the satisfaction/ confidence level of the respondents (Table 5). Overweight respondents had a two-times higher odds ratios (OR = 2.12, 95%CI: 1.77–2.55, p<0.001), respondents with obesity had five-times higher odds ratios (OR = 5.74, 95%CI: 4.76–6.93, p<0.001) of being dissatisfied in comparison to underweight participants. Respondents with PCOS had 1.63 higher odds of being dissatisfied as compared to women without PCOS respondents (OR = 1.63, 95%CI: 1.50–1.77, p<0.001). Similarly, respondents with a rounded body had three times higher odds of dissatisfaction among the respondents (OR = 3.14, 95%CI: 2.5–3.8, p< 0.001). The adjusted model showed similar trends.

**Table 5:** Binary logistics regression analysis assessing whether PCOS, high BMI and body shape had impact on satisfaction/confidence level of the participants.

	Unadjusted Odds ratio	95% CI	p value	Adjusted Odds ratio	95% CI	p value
BMI Underweight Normal Overweight Obesity	0.87 2.12 5.74	0.73–1.05 1.77–2.55 4.76–6.93	0.15 **<0.001** **<0.001**	0.872 1.991 4.963	0.72–1.04 1.65–2.39 4.09–6.01	0.143 **<0.001** **<0.001**
PCOS Yes No (Ref)	1.63	1.50–1.77	<0.001	1.431	1.31–1.56	**<0.001**
Body Shape Triangle Rounded Rectangle Hourglass	1.17 3.14 0.94 0.825	1.0-1.38 2.5–3.80.79–1.110.69–0.9	0.047 **<0.001** 0.49 **0.013**	0.981 1.541 0.955 0.763	0.82–1.16 1.25–1.9 0.8–1.14 0.64–0.9	0.827 **<0.001** 0.611 **0.002**

## Discussion

This study has shown that women with PCOS reported being significantly less confident or content with their bodies than women without PCOS. Women with PCOS were more likely to avoid to social interactions and express feelings of low self-worth and lack of attractiveness. Weight significantly influenced self-value, more in women with PCOS than those women without PCOS, with more women with PCOS wanting to lose weight than those women without PCOS. Furthermore, significantly fewer women with PCOS felt confident/satisfied in their physical appearance. The increased prevalence of women avoiding social interactions, linking appearance to self-worth, feeling unattractive, avoiding social events, avoiding mirrors, and wishing to lose weight within the PCOS population raise concerns about the mental well-being of this patient population, highlighting the need to pay attention to the mental aspects of this syndrome while treating women with PCOS. Symptoms such as social withdrawal and putting great emphasis on appearance may predispose this patient group to psychiatric sequalae such as depression, disordered eating, and body dysmorphia if left unaddressed [23,24]. Such complications would add to the burden of the syndrome on the health of patients as well as health-care systems.

Body image is a complex concept that encompasses a person’s perception of their body regarding their appearance, size, health, normal functioning, as well as their feelings, thoughts, and actions, regarding their own body and appearance [9,10]. Many women with PCOS reported that they tended to avoid their reflection in the mirror and agreed that weight had significantly influenced their self-worth and value, feeling less attractive and avoiding social interactions, which is in accord with the available literature [25,26] and systematic review on body image [7], indicating that the facets and features of PCOS contribute negatively to body image for these women.

Our study revealed that 65.2% PCOS subjects reported being overweight or obese, significantly more than non-PCOS subjects. Obesity has been linked to depression in the general female population, and around two thirds of women with PCOS are overweight or obese [27], in accord with the study findings here where increasing obesity led to greater dissatisfaction that was in accord with a meta-analysis showing lower scores in the weight subscale for the Body Esteem Scale for Adolescents and Adults [7], and reflected those in the lived experience study in PCOS where feeling less feminine was in part related to weight gain [8]. Obesity has been shown to increase the likelihood of losing self-esteem and developing a negative body image, which can lead to lower quality of life and psychological morbidity in addition to being a risk factor for other health issues [28]. In the underweight groups there were no differences between women with PCOS and women without PCOS, though the numbers were very small; however, in the normal weight group, women with PCOS scored significantly lower on parameters of body image satisfaction and confidence indicating that in the absence of obesity, PCOS features have independent effects that are then exacerbated by the obesity as seen by the increased odds ratios for dissatisfaction as weight increased. This was supported by the association of women with PCOS with acne, clinical hirsutism and hair loss being more dissatisfied in comparison to women without PCOS. Hirsutism in particular can negatively affect women’s mental health and cause a deterioration in their social life [29] and both hirsutism and acne have greater body dissatisfaction than healthy control women [30], and in accord with a PCOS study that showed a link between self-image and the severity of depressive symptoms irrespective of BMI [31]. These data are in accord with A population based cohort compared self-reported PCOS versus women who had unrecognized PCOS versus women without PCOS in the Coronary Artery Risk Development in Young Adults (CARDIA) study: those who were self-reporting PCOS had many of the features associated with PCOS such as obesity and diabetes suggesting again that self-reporting of PCOS was consistent with the recognized phenotype [32].

Other important features of PCOS, such as infertility, can also have an impact on self-esteem and confidence. A study conducted in 2006 in Pakistan on 100 women facing difficulties conceiving found that there was a higher prevalence of depression, anxiety, and stress in comparison to the control group [33]. Similarly, another study conducted in Iran reported that infertility, irregular menstruation, and obesity had a negative body image score more than women who were fertile, had normal menstruation, or had a normal BMI [34]. A Qatari study reported how difficulty in conceiving impacted on social life [35]; however, in this study there was no difference between those that responded with PCOS and those that did not.

The findings in the general systems’ questionnaire revealed an increase in metabolic/endocrine and gynecological problems for the PCOS respondents, with no differences for respiratory, cardiac, dermatological, rheumatological and orthopedic problems. The women without PCOS respondents reported higher hematological problems consisting of anemia. It was a surprise to see there were no differences in psychiatric issues as it may have been expected that the PCOS respondents would had a higher incidence [36]. However, this may be explained by the high rate of stigmatism for psychiatric issues in this population [37].

A study in Turkey did not find any difference in the Body Image Scale (BIS) and Rosenberg Self-Esteem Scale (RSES) scores of the women with PCOS women and those women without PCOS in terms of body image or self-esteem [27], that is the converse to that reported here. After controlling for age, dissatisfaction with body appearance was reported to be an independent predictor of depression in women with PCOS [30]. Research on body image and self-esteem has focused on women in the West, and information on Arab populations on this topic has been scarce, hence the importance of this study.

Of the 12,199 respondents, 3329 (27.3%) were women with PCOS, and clearly there was a disparity in how they had the differing facets of PCOS. This in part may reflect that the Rotterdam consensus defines four phenotypes with phenotype A comprising hyperandrogenemia, menstrual irregularity and polycystic ovaries on ultrsound having the most metabolic phenotype [38]. Overall, the prevalence of hirsutism is 70–80% in PCOS patients while the prevalence of acanthosis nigricans, indicative of insulin resistance is 22–30%; therefore, the potential impact of PCOS on self-esteem and body-image satisfaction might be more attributed to more overt manifestations than others [39,40]. PCOS has been associated with depression and reduced QoL in several studies [41,42], it is not yet clear which of its components has the main effect, though obesity is associated with reduced Qol in its own right [43].

This disparity can also be significantly influenced by the role of social media in spreading information regarding PCOS. Previous data has shown the magnitude of social media’s influence and its influencers on perceptions and views of PCOS, with influencers who drive the online content on PCOS significantly affecting behaviour and decision making for patients living with PCOS [11,12]. Data has also shown a significant disparity in views on PCOS between women and men, with the latter being unfortunately associated with non-evidence-based approaches and misinformation [11]. Additionally, there is a significant global inequity and misrepresentation in sources of information on PCOS, with over 80% of individuals promoting this content being only from high-income countries [12].

The results of this study have implications for clinical practice and suggest that an educational/ counselling program delivered to woman with PCOS by a multidisciplinary team will have a positive impact on their emotional well-being. One of the non-pharmacological ways to improve women with PCOS body image and self-esteem is to use acceptance and commitment therapy (ACT) frequently because of its positive effects on their mental health [12]. Explaining the relationship between side effects of PCOS and its symptoms may be useful in alleviating their negative influence on self-esteem. A shift in perspective towards viewing their body results from treatment utilising cognitive techniques, which include establishing personal values during sessions, improving acceptance, and minimizing emotional flaws. Screening women with PCOS who are deemed at risk (i.e., obese or facing difficulties conceiving) by their multidisciplinary team responsible for their management plan should be done for possible complication such as disordered eating or depressive tendencies to prevent a further physical and psychological burden on the patient and their quality of life. This highlights the need to screen for body image concerns in the clinical setting with the use of with the use of the Body Image Concern Inventory (BICI) [44] and the Rosenberg Self-Esteem Scale [45].

The strength of this study was the large number of respondents. Limitations include the lack of confirmation of PCOS respondents through medical record review; however, this limitation regarding the diagnosis of PCOS and grouping together of women meeting different PCOS criteria is also present in consortia that rely on electronic medical records, which include not only diagnoses by healthcare professionals but also specific symptom criteria of PCOS [32]. Additionally, limitations of the study include the method of sampling used that was nonprobability sampling, known as convenience sampling [46], used in cross-sectional studies, and could lead to sampling bias due to a younger demographic using social media. However, this was mitigated in this study as the population being targeted is the younger reproductive population, with mean age of respondents at 31-years-old. While the use of social media platforms has the advantage of reaching a large sample, it may be biased as a different demographic may be using other different platforms and may not reach the larger MENA population. Future social media studies may benefit in including more platforms and to determine if there are differences in the responses. The use of an adapted questionnaire poses another limitation on the study as this specific questionnaire used was not validated; however, this was designed to be appropriate and comprehendible when translated to Arabic, the native language of the MENA region. Recent studies are investigating the strengths and limitation of online platforms as a tool of data collection, and comparing it to traditional data collection methods [47]. A study by the IOSR Journal of Humanities and Social Sciences looked into the strengths and weaknesses of online questionnaires [48]. The study found that online questionnaires showed comparable results to the traditional questionnaires. In addition, no significant difference was found between online and traditional questionnaires in terms of expression of sensitive data nor accuracy of responses [49]. and the respondents found online questionnaires faster, more relaxing, and more interesting. Online questionnaires were also found to lower social desirability bias and item nonresponse, making the collected data of higher value as well as increase anonymity. This mode of data collection is most suitable for descriptive, cohort, evaluation, and case-control studies. The disadvantages found were mainly regarding how comfortable the respondents were using different social media platforms, and the response rate, which was shown to be higher in traditional questionnaires in comparison to online ones. However, with the tremendous reach social media holds, the low response rate can be overcome with marketing strategies and strategic sharing of the questionnaire to an interested target audience. Lastly, since the Middle Eastern region is known to host a large expatriate community, and given that previous data suggest emotional and psychosexual well-being being significantly influenced by ethnicity and birthplace of women with PCOS [50], an additional limitation of the study is its lack of assessment of ethnicity or birthplace of participants. Future work will aim at including this variable in all analyses and adjusting for it to discern any differences.

It is of major importance that the 2023 international PCOS guidelines include body image as a new recommendation for the assessment in PCOS management [6], and a systematic review that is in accord with the findings of this study, has highlighted the body image concerns that women with PCOS have [7]. However, there is the need for future research that should focus on defining the issue of body image through validated questionnaires that include emotional and psychosexual elements and address ethnicity [51]. There is a limited literature on PCOS models of care; therefore, there is a need to explore new models of care in alignment with international guidelines, potentially facilitated through the exchange of information between institutions with established best practice [52].

## Conclusion

PCOS respondents reported significantly lower measures of self-esteem and body image satisfaction compared to non-PCOS respondents in this population, with a significant difference in the number of PCOS women having obesity, a rounded (metabolic syndrome) body shape, hirsutism, and acanthosis nigricans indicative of insulin resistance. It is critical that these issues are addressed through focused multidisciplinary programs, with future research to address body image across differing ethnicities.

## Supporting information

S1 Fig Box and whisker plot comparison of body mass index (BMI) between women without PCOS (n = 8870) versus those reporting PCOS (n = 3329). (TIF)

S1 File Full questionnaire used for study in Arabic and English version. Data set consisting of the data required to replicate all study findings.
(DOCX)

S1 Raw data (XLSX)

## Abbreviations

ACT: Acceptance and Commitment therapy (ACT)
BAS-2: Body Appreciation Scale-2
BIS: Body Image Scale
BMI: Body Mass Index
CDC: Centres for Disease Control and Prevention
EDE-Q 6.0: Eating Disorder Examination Questionnaire
MENA: Middle East and North Africa
NIH: National Institute of Health
PCOS: Polycystic ovary syndrome
QoL: Quality of Life
RSES: Rosenberg Self-Esteem Scale
T2D: Type 2 diabetes
WHO: World Health Organization
