# Supplementary figures and images for: Self-esteem and body image satisfaction in women with PCOS in the Middle East: Cross-sectional social media study
Source: PLoS One. 2024 Apr 25;19(4):e0301707. doi: 10.1371/journal.pone.0301707 (PMC11045070; doi:10.1371/journal.pone.0301707)

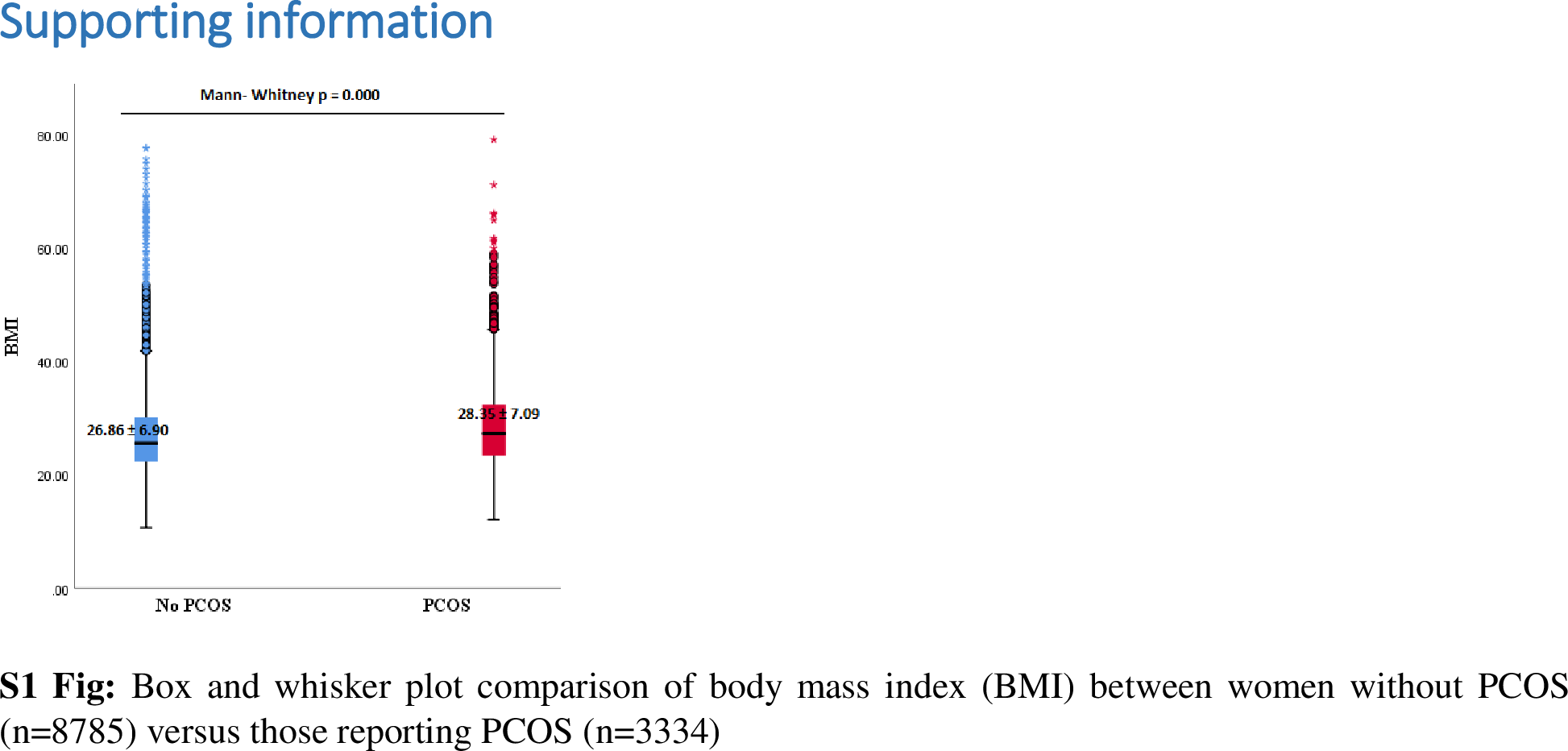

Supplement: S1 Fig — (TIF) [file pone.0301707.s001.tif]
