# Supplementary material for: Self-esteem and body image satisfaction in women with PCOS in the Middle East: Cross-sectional social media study
Source: PLoS One. 2024 Apr 25;19(4):e0301707. doi: 10.1371/journal.pone.0301707 (PMC11045070; doi:10.1371/journal.pone.0301707)
Supplement: S1 File — Data set consisting of the data required to replicate all study findings. (DOCX) [file pone.0301707.s002.docx]

Full Questionnaire used:

1. Gender:

- Male
- Female

1. Age:
2. Weight (kg):
3. Height (cm):


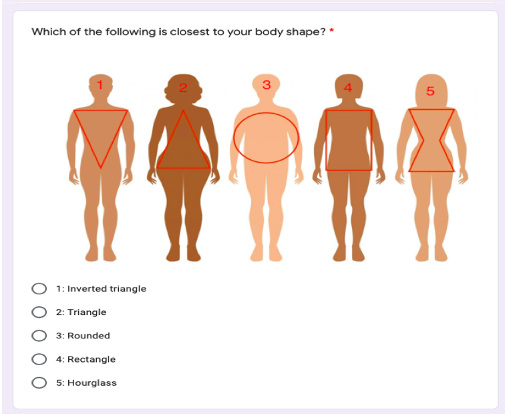


1. Marital Status:

- Single
- Married
- Widowed
- Divorced

1. Do you suffer from any health problems:

- Polycystic ovarian syndrome (PCOS)
- Type 1 diabetes
- Type 2 diabetes
- Prediabetes
- High prolactin levels
- Thyroid issues
- Ovarian insufficiency
- High blood pressure (hypertension)
- High blood cholesterol
- Gout
- Other:

1. Are you currently facing difficulties conceiving:

- Yes
- No
- I am not trying to conceive

1. Do you currently suffer from irregular or missed periods (Less than 9 menstrual periods per year or longer than 35 days between periods or shorter than 21 days between periods):

- Yes, my period is irregular
- No, my period is regular

1. Do you currently have hirsutism (excessive thick coarse hair growth in areas such as the neck, face, abdomen, chest, and thighs)

- Yes
- No

1. Do you currently suffer from acne (on the face or body):

- Yes
- No

1. Do you currently experience male-pattern hair loss (receding hairline, hair loss on the crown of the head):

- Yes
- No

1. Have you currently noticed dry, dark patches of skin in areas such as the neck, armpits, thighs, or groin:

- Yes
- No

1. Has your body shape or weight influenced how you think about yourself as a person? (i.e. feeling disappointed after weight gain or feeling proud after weight loss):

- Yes
- No

1. Does it sometimes upset you or do you avoid seeing yourself in the mirror/ taking pictures of yourself:

- Yes
- No

1. Do you agree with the following statement: “I sometimes spend long times across the mirror analyzing my appearance”:

- Agree
- Disagree

1. Do you agree with the following statement: “I feel attractive”:

- Agree
- Disagree

1. Do you agree with the following statement: “I sometimes avoid social interactions because of my weight/appearance or the fear of being judged for the way I look”:

- Agree
- Disagree

1. In regards to your current weight, which of the following do you agree to the most:

- I would like to lose weight
- I would like to maintain my current weight
- I would like to gain weight

1. Do you agree with the following statement: “I am satisfied and confident with the way I look”?

- Agree
- Disagree

الاستبيان:

1. الجنس:

- ذكر
- انثى

1. العمر:
2. الطول (سم):
3. الوزن (كغ):


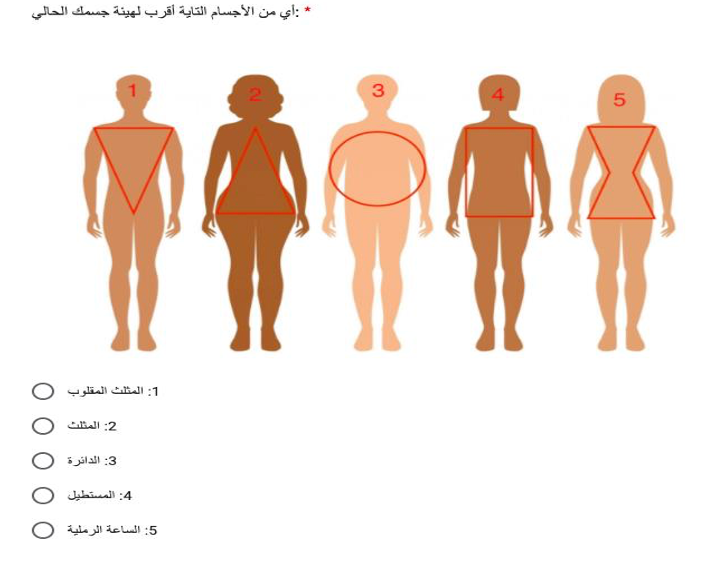


٦. الحالة الاجتماعية:

عزباء -

- متزوجة

- ارملة

- مطلقة

٧.هل تعانين من أي مشاكل صحية مما يلي:

- متلازمة تكييس المبايض

- ارتفاع بهرمون الحليب

- فشل المبايض المبكر

- السكري من النوع الأول

- السكري من النوع الثاني

- مقدمات السكري

- ارتفاع بضغط الدم

- ارتفاع بكلسترول الدم

- النقرس

- مشاكل بالغدة الدرقية

- أخرى:

٨. هل تواجهين صعوبة في الانجاب حالياً:

- نعم

- لا

- لا أحاول الانجاب

٩. هل تعانين حالياً من عدم انتظام الدورة الشهرية (أقل من تسعة دورات في السنة أو أكثر من ٣٥ يوم بين الدورتين أو أقل من ٢١ يوم بين الدورتين):

- نعم، الدورة الشهرية لدي غير منتظمة

- لا، الدورة الشهرية لدي منتظمة

١٠. هل تعانين حالياً من الشعرانية أو نمو شعر داكن أو غليظ في منطقة الوجه/الصدر/الرقبة/البطن/الأرداف/الأفخاذ:

- نعم

- لا

١١. هل تعانين حالياً من حب الشباب (على الوجه أو الجسم):

- نعم

- لا

١٢. هل تعانين حالياً من الصلع الذكوري أو تساقط الشعر بشكل ملاحظ عند منبت الشعر أو أعلى فروة الرأس:

- نعم

- لا

١٣. هل لاحظتي حالياً تشققات أو اسوداد في البشرة عند منطقة الرقبة أو الابطين أو المنطقة الحساسة:

- نعم

- لا

١٤. هل يؤثر مظهرك أو وزنك على نظرتك لذاتك أو رضاك عن ذاتك (على سبيل المثال الإحساس بالرضى عند خسارة الوزن أو الإحساس بخيبة الأمل عند اكتساب بعض الوزن)؟

- نعم، يؤثر مظهري أو وزني على نظرتي لذاتي

- لا، لا يوجد تأثير لمظهري على نظرتي لذاتي

١٥. هل توافقين الرأي مع الجملة التالية: رؤية انعكاسي في المرآة أو مظهري في الصور يسبب لي احباط:

- نعم، أوافق

- لا، لا أوافق

١٦. هل تقضين وقتاً مطوّلاً تنظرين إلى مظهرك في المرآة وتحللين تفاصيل مظهرك (خصوصاً التفاصيل التي لا تعجبك):

- نعم

- لا

١٧. هل توافقين الرأي مع الجملة التالية: أؤمن أنني جذابة وجميلة:

- نعم، أوافق

- لا، لا أوافق

١٨. هل توافقين الرأي مع الجملة التالية: أتجنب المناسبات والفعاليات الاجتماعية بسبب عدم ارتياحي أو خجلي من مظهري أو وزني:

- نعم، أوافق

- لا، لا أوافق

١٩. بالنسبة لوزنك الحالي، تودين:

- تنزيل وزنك الحالي

- المحافظة على وزنك الحالي

- زيادة وزنك الحالي

٢٠.هل توافقين الرأي مع الجملة التالية: أشعر بالرضى والراحة من مظهري الحالي:

- نعم، أوافق

- لا، لا أوافق
